# Supplementary material for: ESCRT-III-dependent adhesive and mechanical changes are triggered by a mechanism detecting alteration of septate junction integrity in Drosophila epithelial cells
Source: eLife. 2024 Feb 2;13:e91246. doi: 10.7554/eLife.91246 (PMC10959524; doi:10.7554/eLife.91246)
Supplement: MDAR checklist [file elife-91246-mdarchecklist1.docx]

**Materials Design Analysis Reporting (MDAR)**

**Checklist for Authors**

The [MDAR framework](https://osf.io/xfpn4/) establishes a minimum set of requirements in transparent reporting mainly applicable to studies in the life sciences.

*eLife* asks authors to **provide detailed information within their article** to facilitate the interpretation and replication of their work. Authors can also upload supporting materials to comply with relevant reporting guidelines for health-related research (see [EQUATOR Network](http://www.equator-network.org/%20)), life science research (see the [BioSharing Information Resource](http://biosharing.org/)), or animal research (see the [ARRIVE Guidelines](http://www.plosbiology.org/article/info:doi/10.1371/journal.pbio.1000412) and the [STRANGE Framework](https://doi.org/10.1038/d41586-020-01751-5); for details, see *eLife*’s [Journal Policies](https://reviewer.elifesciences.org/author-guide/journal-policies)). Where applicable, authors should refer to any relevant reporting standards materials in this form.

For all that apply, please note **where in the article** the information is provided. Please note that we also collect information about data availability and ethics in the submission form.

**Materials:**

| **Newly created materials** | **Indicate where provided: section/figure legend** | **N/A** |
| --- | --- | --- |
| The manuscript includes a dedicated "materials availability statement" providing transparent disclosure about availability of newly created materials including details on how materials can be accessed and describing any restrictions on access. | N/A |  |
|  |  |  |
| **Antibodies** | **Indicate where provided: section/figure legend** | **N/A** |
| For commercial reagents, provide supplier name, catalogue number and [RRID](https://scicrunch.org/resources), if available. | **Figure 1D:**  Phalloidin: Alexa Fluor^TM^ 647 Phalloidin (Thermo Fisher Scientific; Cat#A22287)  **Figure 2A:**  pMyo-II: Anti phospho-MyoII (Mouse, monoclonal; Cell Signalling; Cat#mab3675)  **Figures 3A-B-B’:**  Shrub:.GFP: Anti-GFP (Goat, polyclonal; Abcam; Cat#ab5450)  HRS: Anti-HRS (Mouse, monoclonal; DSHB; Cat#27-4)  **Figure 3A’:**  Nrx-IV: Anti-Nrx-IV (Rabbit, polyclonal; Gift from Christian Klämbt)  **Figures 3C-D-D’:**  Shrub::GFP: Anti-GFP (Goat, polyclonal; Abcam; Cat#ab5450)  HRS: Anti-HRS (Mouse, monoclonal; DSHB; Cat#27-4)  **Figure 3C’:**  Nrx-IV: Anti-Nrx-IV (Rabbit, polyclonal; Gift from Christian Klämbt)  **Figure 4A:**  FK2: Anti-FK2 (Mouse, monoclonal; Sigma Aldrich; Cat#04-263)  Nrx-IV: Anti-Nrx-IV (Rabbit, polyclonal; Gift from Christian Klämbt)  **Figures 4B-D:**  Shrub::GFP: Anti-GFP (Goat, polyclonal; Abcam; Cat#ab5450)  FK2: Anti-FK2 (Mouse, monoclonal; Sigma Aldrich; Cat#04-263)  Nrx-IV: Anti-Nrx-IV (Rabbit, polyclonal; Gift from Christian Klämbt)  **Figure 4E:**  FK2: Anti-FK2 (Mouse, monoclonal; Sigma Aldrich; Cat#04-263)  **Figure 5B:**  Crb antibody: Anti-Crb (Rat, polyclonal; Gift from Elisabeth Knust)  **Figures 6A-B:**  Phalloidin: Alexa Fluor^TM^ 647 Phalloidin (Thermo Fisher Scientific; Cat#A22287)  Mys: Anti-Mys (Rabbit, monoclonal; DSHB; CF.6G1; Cat#AB_528310)  **Figures 6C-D:**  Mys: Anti-Mys (Rabbit, monoclonal; DSHB; CF.6G1; Cat#AB_528310)  **Figures 6E-E’:**  E-Cad:Anti-E-cad (Rat, monoclonal; DSHB; DCAD2; Cat#AB_528120)  Mys: Anti-Mys (Rabbit, monoclonal; DSHB; CF.6G1; Cat#AB_528310)  **Figure S1B:**  E-Cad:Anti-E-cad (Rat, monoclonal; DSHB; DCAD2; Cat#AB_528120)  **Figures S3A-C’:**  HRS: Anti-HRS (Mouse, monoclonal; DSHB, 27-4)  **Figures S4A-B’’’’:**  Kune: Anti-Kune (Rabbit, polyclonal ; Gift from Mikio Furuse)  Crb antibody: Anti-Crb (Rat, polyclonal; Gift from Elisabeth Knust)  **Figures S5B-B’:**  Crb antibody: Anti-Crb (Rat, polyclonal; Gift from Elisabeth Knust)  **Figures S6A-A’’’’:**  Kune: Anti-Kune (Rabbit, polyclonal ; Gift from Mikio Furuse)  Mys: Anti-Mys (Rabbit, monoclonal; DSHB; CF.6G1; Cat#AB_528310) |  |
|  |  |  |
|  |  |  |
| **DNA and RNA sequences** | **Indicate where provided: section/figure legend** | **N/A** |
| Short novel DNA or RNA including primers, probes: Sequences should be included or deposited in a public repository. | N/A |  |
|  |  |  |
| **Cell materials** | **Indicate where provided: section/figure legend** | **N/A** |
| Cell lines: Provide species information, strain. Provide accession number in repository OR supplier name, catalog number, clone number, OR RRID. | N/A |  |
| Primary cultures: Provide species, strain, sex of origin, genetic modification status. | N/A |  |
|  |  |  |
| **Experimental animals** | **Indicate where provided: section/figure legend** | **N/A** |
| Laboratory animals or Model organisms: Provide species, strain, sex, age, genetic modification status. Provide accession number in repository OR supplier name, catalog number, clone number, OR RRID. | **Figure 1:** *Drosophila melanogaster,* pupal stage  (A–A′) UAS-Aka-RNAi-TRiP ; *pnr*-Gal4 obtained by crossing UAS-Aka-RNAi-TRiP with *pnr*-Gal4 / TM6, *Tb1*  (B–B′) *hs*-FLP ; *aka^L200^*, FRT40A / *ubi*-RFP nls, FRT40A, E-cad::GFP ; obtained by crossing *hs*-FLP ; *aka^L200^*, FRT40A / CyO with *hs*-FLP ; *ubi*-RFP nls, FRT40A, ECad::GFP / CyO  (C–C′) *hs*-FLP ; Myo-II::GFP; *ubi*-RFP nls, FRT40A / CyO obtained by crossing Myo-II::GFP; *ubi*-RFP nls, FRT40A / CyO ; with *hs*-FLP ; *aka^L200^*, FRT40A / CyO  (D–D′) *hs*-FLP ; *aka^L200^*, FRT40A / *ubi* -RFP nls, FRT40A obtained by crossing *hs*-FLP; *aka^L200^*, FRT40A / CyO with *hs*-FLP ; *ubi*-RFP nls, FRT40A / (CyO)  (E–E′) *hs*-FLP ; *nrv2^k13315^*, FRT40A / *ubi* -RFP nls, FRT40A, E-cad::GFP ; obtained  by crossing *hs*-FLP ; *nrv2^k13315^*, FRT40A / CyO with *hs*-FLP ; *ubi*-RFP nls, FRT40A,  E-cad::GFP / CyO  (F-F’) *hs*-FLP / Myo-II::GFP ; *nrv2^k13315^*, FRT40A / *ubi*-RFP nls, FRT40A obtained by crossing Myo-II::GFP; *ubi*-RFP nls, FRT40A / CyO ; with *hs*-FLP ; *nrv2^k13315^*,FRT40A  / CyO  (G-G’) *hs*-FLP ; *cold^f05607^*, FRT40A / *ubi*-RFP nls, FRT40A, E-cad::GFP ; obtained by crossing *hs*-FLP ; *cold^f05607^*, FRT40A / CyO with *hs*-FLP ; *ubi*-RFP nls, FRT40A, ECad::GFP / CyO  **Figure 2:** *Drosophila melanogaster,* pupal stage  (A-B) *hs*-FLP ; Myo-II::GFP; *ubi*-RFP nls, FRT40A / CyO obtained by crossing Myo-II::GFP; *ubi*-RFP nls, FRT40A / CyO ; with *hs*-FLP ; *aka^L200^*, FRT40A /  CyO  (E-F) *hs*-FLP ; *aka^L200^*, FRT40A / *ubi*-RFP nls, FRT40A, E-cad::GFP ; obtained by crossing *hs*-FLP ; *aka^L200^*, FRT40A / CyO with *hs*-FLP ; *ubi*-RFP nls, FRT40A, ECad::GFP / CyO  **Figure 3:** *Drosophila melanogaster,* pupal stage  (A-D’) Shrub::GFP ; UAS-cora-RNAi / UAS-KAEDE, *pnr*-Gal4 obtained by crossing UAS-cora-RNAi with ; Shrub::GFP ; UAS-KAEDE, *pnr*-Gal4 / SM5-TM6, *Tb1*  **Figure 4:** *Drosophila melanogaster,* pupal stage  (A) *sca*-Gal4 / UAS-Shrub-RNAi-TRiP obtained by crossing ;; *sca*-Gal4 with ;; UAS-Shrub-RNAi-TRiP  (B–C’’’) Shrub::GFP ; UAS-cora-RNAi /*pnr*-Gal4 obtained by crossing UAS-cora-RNAi  with ; Shrub::GFP ; *pnr*-Gal4 / SM5-TM6, *Tb1*  (D–D′′) Shrub::GFP ; UAS-Nrx-IV-RNAi / *pnr*-Gal4 obtained by crossing UAS-Nrx-IV-RNAi with Shrub::GFP ; *pnr*-Gal4 / TM6, *Tb1*  (E) *hs*-FLP ; *aka^L200^*, FRT40A / *ubi*-RFP nls, FRT40A obtained by crossing *hs*-FLP;  *aka^L200^*, FRT40A / CyO with *hs*-FLP ; *ubi*-RFP nls, FRT40A / (CyO)  **Figure 5:** *Drosophila melanogaster,* pupal stage  (A–A′) *hs*-FLP ; *aka^L200^*, FRT40A / *ubi*-RFP nls, FRT40A ; Crb::GFP / + obtained by  crossing *ubi*-RFP nls, FRT40A / CyO ; Crb::GFP / TM6, *Tb1* with *hs*-FLP ; *aka^L200^*,  FRT40A / CyO  (B–B′) *hs*-FLP ; *aka^L200^*, FRT40A / *ubi* -RFP nls, FRT40A obtained by crossing *hs*-FLP ; *aka^L200^*, FRT40A / CyO with *hs*-FLP ; *ubi*-RFP nls, FRT40A / (CyO)  (D-D’) *hs*-FLP ; *aka^L200^*, FRT40A / *ubi*-RFP nls, FRT40A, E-cad::GFP ; FRT82B, Crb^11a22^ / + obtained by crossing ; *aka^L200^*, FRT40A / CyO ; FRT82B, Crb^11a22^ / TM6, *Tb1* with *hs*-FLP ; *ubi*-RFP nls, FRT40A, ECad::GFP / CyO  **Figure 6:** *Drosophila melanogaster,* pupal stage  (A–D) *hs*-FLP ; *aka^L200^*, FRT40A / *ubi* -RFP nls, FRT40A obtained by crossing *hs*-FLP ; *aka^L200^*, FRT40A / CyO with *hs*-FLP ; *ubi*-RFP nls, FRT40A / (CyO)  (E-E’) *hs*-FLP, UAS-GFP; *aka^L200^*, FRT40A / *tub*-GAL80, FRT40A; UAS-Mys-RNAi-TRiP/ *tub*-GAL4 obtained by crossing *aka^L200^*, FRT40A; UAS-Mys-RNAi-TRiP /SM5-TM6b, *Tb1* with *hs*-FLP, UAS-GFP, *tub*-GAL80, FRT40A; *tub*-GAL4 /TM6C, *Sb_1_*, *Tb1*  **Figure S1:** *Drosophila melanogaster,* pupal stage  (A–A′′) *hs*-FLP ; *aka^L200^*, FRT40A / *ubi*-RFP nls, FRT40A, E-cad::GFP ; obtained by crossing *hs*-FLP ; *aka^L200^*, FRT40A / CyO with *hs*-FLP ; *ubi*-RFP nls, FRT40A, ECad::GFP / CyO  (B) *hs*-FLP ; *Gli^dv3^*, FRT40A / *ubi*-RFP nls, FRT40A ; obtained by crossing *hs*-FLP ; *Gli^dv3^*, FRT40A / CyO with *hs*-FLP ; *ubi*-RFP nls, FRT40A / CyO  (C-C’) *hs*-FLP ; *aka^L200^*, FRT40A / *ubi*-RFP nls, FRT40A, E-cad::GFP ; obtained by crossing *hs*-FLP ; *aka^L200^*, FRT40A / CyO with *hs*-FLP ; *ubi*-RFP nls, FRT40A, ECad::GFP / CyO  **Figure S2:** *Drosophila melanogaster,* pupal stage  (A–B) *hs*-FLP ; *aka^L200^*, FRT40A / *ubi*-RFP nls, FRT40A ; Vinc::GFP / + obtained by  crossing *ubi*-RFP nls, FRT40A / CyO ; Vinc::GFP / TM6, *Tb1* with *hs*-FLP ; *aka^L200^*,  FRT40A / CyO  (D–D′) *hs*-FLP ; *aka^L200^*, FRT40A / *ubi*-RFP nls, FRT40A ; Karst::YFP / + obtained by  crossing ; *ubi*-RFP nls, FRT40A / CyO ; Karst::YFP / TM6, *Tb1* with *hs*-FLP ; *aka^L200^*,  FRT40A / CyO  (E-E’) *hs*-FLP ; *aka^L200^*, FRT40A / *ubi*-RFP nls, FRT40A ; Jub::GFP / + obtained by  crossing ; *ubi*-RFP nls, FRT40A / CyO ; Jub::GFP / TM2 with *hs*-FLP ; *aka^L200^*, FRT40A / CyO  **Figure S3:** *Drosophila melanogaster,* pupal stage  (A–A′) *hs*-FLP ; *aka^L200^*, FRT40A / *ubi* -RFP nls, FRT40A obtained by crossing *hs*-FLP ; *aka^L200^*, FRT40A / CyO with *hs*-FLP ; *ubi*-RFP nls, FRT40A / (CyO)  (C–C′) *hs*-FLP ; *nrv2^k13315^*, FRT40A / *ubi* -RFP nls, FRT40A ; obtained by crossing *hs*-FLP ; *nrv2^k13315^*, FRT40A / CyO with *hs*-FLP ; *ubi*-RFP nls, FRT40A / CyO  **Figure S4:** *Drosophila melanogaster,* pupal stage  (A-B’’’’) *sca*-Gal4 / UAS-Shrub-RNAi-TRiP obtained by crossing ;; *sca*-Gal4 with ;; UAS-Shrub-RNAi-TRiP  **Figure S5:** *Drosophila melanogaster,* pupal stage  (A–A′) *hs*-FLP ; *nrv2^k13315^*, FRT40A / *ubi*-RFP nls, FRT40A ; Crb::GFP / + obtained by crossing ; *ubi*-RFP nls, FRT40A / CyO ; Crb::GFP / TM6, *Tb1* with *hs*-FLP ; *nrv2^k13315^*, FRT40A / CyO  (B–B′) *hs*-FLP ; *nrv2^k13315^*, FRT40A / *ubi* -RFP nls, FRT40A ; obtained by crossing *hs*-FLP ; *nrv2k13315*, FRT40A / CyO with *hs*-FLP ; *ubi*-RFP nls, FRT40A / CyO  **Figure S6:** *Drosophila melanogaster,* pupal stage  (A-A’’’’) *sca*-Gal4 / UAS-Shrub-RNAi-TRiP obtained by crossing ;; *sca*-Gal4 with ;; UAS-Shrub-RNAi-TRiP |  |
| Animal observed in or captured from the field: Provide species, sex, and age where possible. | N/A |  |
|  |  |  |
| **Plants and microbes** | **Indicate where provided: section/figure legend** | **N/A** |
| Plants: provide species and strain, ecotype and cultivar where relevant, unique accession number if available, and source (including location for collected wild specimens). | N/A |  |
| Microbes: provide species and strain, unique accession number if available, and source. | N/A |  |
|  |  |  |
| **Human research participants** | **Indicate where provided: section/figure legend) or state if these demographics were not collected** | **N/A** |
| If collected and within the bounds of privacy constraints report on age, sex, gender and ethnicity for all study participants. | N/A |  |

**Design:**

| **Study protocol** | **Indicate where provided: section/figure legend** | **N/A** |
| --- | --- | --- |
| If the study protocol has been pre-registered, provide DOI. For clinical trials, provide the trial registration number OR cite DOI. | N/A |  |
|  |  |  |
| **Laboratory protocol** | **Indicate where provided: section/figure legend** | **N/A** |
| Provide DOI OR other citation details if detailed step-by-step protocols are available. | N/A |  |
|  |  |  |
| **Experimental study design (statistics details) *** | | |
| **For in vivo studies: State whether and how the following have been done** | **Indicate where provided: section/figure legend. If it could have been done, but was not, write “not done”** | **N/A** |
| Sample size determination | N/A |  |
| Randomisation | N/A |  |
| Blinding | N/A |  |
| Inclusion/exclusion criteria | N/A |  |
|  |  |  |
| **Sample definition and in-laboratory replication** | **Indicate where provided: section/figure legend** | **N/A** |
| State number of times the experiment was replicated in the laboratory. | Figures 1A-A’’: 2 replicates  Figures 1B-B’: 10 replicates  Figures 1C-C’: 5 replicates  Figures 1D-D’: 5 replicates  Figures 1E-E’: 2 replicates  Figures 1F-F’: 2 replicates  Figures 1G-G’: 4 replicates  Figures 2A-A’: > 5 replicates  Figures 2B-D: > 5 replicates  Figures 2E-G: > 5 replicates  Figure 3: > 5 replicates  Figure 4A: 2 replicates  Figures 4B-C’’’: >5 replicates  Figure 4D: 4 replicates  Figure 4E: 3 replicates  Figures 5A-C’: 5 replicates  Figures 5B-C’: >5 replicates  Figures 5D-E: 2 replicates  Figures 6A-B: 4 replicates  Figures 6C-D: 2 replicates  Figures 6E-G: 4 replicates  Figures S1A-A’’: >10 replicates  Figures S1B-B’: 4 replicates  Figures S1C-D: 4 replicates  Figures S1E: >5 replicates  Figures S2A-C: 4 replicates  Figures S2D-D’: 4 replicates  Figures S2E-E’: 2 replicates  Figures S3A-B: 4 replicates  Figures S3C-D: 4 replicates  Figures S4: 2 replicates  Figures S5A-C: 4 replicates  Figures S5B-C: 5 replicates  Figures S6A-A’’’’: 2 replicates |  |
| Define whether data describe technical or biological replicates. | Technical replicate: experience performed  Biological replicate: number of pupae  Figures 1A-A’’: 2 technical replicates; 3 biological replicates  Figures 1B-B’: >10 technical replicates; >10 biological replicates  Figures 1C-C’: 5 technical replicates; 5 biological replicates  Figures 1D-D’: 5 technical replicates; 5 biological replicates  Figures 1E-E’: 2 technical replicates; 2 biological replicates  Figures 1F-F’: 2 technical replicates; 2 biological replicates  Figures 1G-G’: 4 technical replicates; 4 biological replicates  Figure 2A: >5 technical replicates; >10 biological replicates  Figures 2B-D: >5 technical replicates; >10 biological replicates  Figures 2E-G: >5 technical replicates; >10 biological replicates  Figure 3: >5 technical replicates; >5 biological replicates  Figure 4A: 2 technical replicates; 3 biological replicates  Figures 4B-C’’’: >5 technical replicates; >5 biological replicates  Figures 4D: 4 technical replicates; >5 biological replicates  Figures 4E: 3 technical replicates; >5 biological replicates  Figures 5A-C’: 5 technical replicates; 5 biological replicates  Figures 5B-C’: >5 technical replicates; >5 biological replicates  Figures 5D-E: 2 technical replicates; >5 biological replicates  Figures 6A-B: 4 technical replicates; >5 biological replicates  Figures 6C-D: 2 technical replicates; 3 biological replicates  Figures 6E-G: 4 technical replicates; >5 biological replicates  Figures S1A-A’’: >10 technical replicates; >20 biological replicates  Figures S1B-B’: 4 technical replicates; 4 biological replicates  Figures S1C-D: 4 technical replicates; 4 biological replicates  Figures S1E: >5 technical replicates; >5 biological replicates  Figures S2A-C: 4 technical replicates; 4 biological replicates  Figures S2D-D’: 4 technical replicates; 4 biological replicates  Figures S2E-E’: 2 technical replicates; 2 biological replicates  Figures S3A-B: 4 technical replicates; >5 biological replicates  Figures S3C-D: 4 technical replicates; >5 biological replicates  Figures S4: 2 technical replicates; >5 biological replicates  Figures S5A-C: 4 technical replicates; 5 biological replicates  Figures S5B-C: 5 technical replicates; 5 biological replicates  Figures S6A-A’’’: 2 technical replicates; 3 biological replicates |  |
|  |  |  |
| **Ethics** | **Indicate where provided: section/submission form** | **N/A** |
| Studies involving human participants: State details of authority granting ethics approval (IRB or equivalent committee(s), provide reference number for approval. | N/A |  |
| Studies involving experimental animals: State details of authority granting ethics approval (IRB or equivalent committee(s), provide reference number for approval. | N/A |  |
| Studies involving specimen and field samples: State if relevant permits obtained, provide details of authority approving study; if none were required, explain why. | N/A |  |
|  |  |  |
| **Dual Use Research of Concern (DURC)** | **Indicate where provided: section/submission form** | **N/A** |
| If study is subject to dual use research of concern regulations, state the authority granting approval and reference number for the regulatory approval. | N/A |  |

**Analysis:**

| **Attrition** | **Indicate where provided: section/figure legend** | **N/A** |
| --- | --- | --- |
| Describe whether exclusion criteria were pre-established. Report if sample or data points were omitted from analysis. If yes, report if this was due to attrition or intentional exclusion and provide justification. | N/A |  |
|  |  |  |
| **Statistics** | **Indicate where provided: section/figure legend** | **N/A** |
| Describe statistical tests used and justify choice of tests. | No statistical tests were used to predetermine sample size. Replicates numbers were decided from experience of the techniques performed and practical considerations. No data were excluded.  We displayed the means and standard deviations on our graphs.  The Shapiro-Wilk normality test was used to confirm the normality of the data and the F-test to verify the equality of SD.  The statistical difference of data sets was analyzed using the Student unpaired two-tailed t test, Multiple t tests, Fisher t test or the non-parametric Wilcoxon-Mann-Whitney test. |  |
|  |  |  |
| **Data availability** | **Indicate where provided: section/submission form** | **N/A** |
| For newly created and reused datasets, the manuscript includes a data availability statement that provides details for access (or notes restrictions on access). | N/A |  |
| When newly created datasets are publicly available, provide accession number in repository OR DOI and licensing details where available. | N/A |  |
| If reused data is publicly available provide accession number in repository OR DOI, OR URL, OR citation. | N/A |  |
|  |  |  |
| **Code availability** | **Indicate where provided: section/figure legend** | **N/A** |
| For any computer code/software/mathematical algorithms essential for replicating the main findings of the study, whether newly generated or re-used, the manuscript includes a data availability statement that provides details for access or notes restrictions. | N/A |  |
| Where newly generated code is publicly available, provide accession number in repository, OR DOI OR URL and licensing details where available. State any restrictions on code availability or accessibility. | N/A |  |
| If reused code is publicly available provide accession number in repository OR DOI OR URL, OR citation. | N/A |  |

**Reporting:**

The MDAR framework recommends adoption of discipline-specific guidelines, established and endorsed through community initiatives.

| **Adherence to community standards** | **Indicate where provided: section/figure legend** | **N/A** |
| --- | --- | --- |
| State if relevant guidelines (e.g., ICMJE, MIBBI, ARRIVE, STRANGE) have been followed, and whether a checklist (e.g., CONSORT, PRISMA, ARRIVE) is provided with the manuscript. | N/A |  |

* We provide the following guidance regarding transparent reporting and statistics; we also refer authors to [Ten common statistical mistakes to watch out for when writing or reviewing a manuscript](https://doi.org/10.7554/eLife.48175).

**Sample-size estimation**

- You should state whether an appropriate sample size was computed when the study was being designed
- You should state the statistical method of sample size computation and any required assumptions
- If no explicit power analysis was used, you should describe how you decided what sample (replicate) size (number) to use

**Replicates**

- You should report how often each experiment was performed
- You should include a definition of biological versus technical replication
- The data obtained should be provided and sufficient information should be provided to indicate the number of independent biological and/or technical replicates
- If you encountered any outliers, you should describe how these were handled
- Criteria for exclusion/inclusion of data should be clearly stated
- High-throughput sequence data should be uploaded before submission, with a private link for reviewers provided (these are available from both GEO and ArrayExpress)

**Statistical reporting**

- Statistical analysis methods should be described and justified
- Raw data should be presented in figures whenever informative to do so (typically when N per group is less than 10)
- For each experiment, you should identify the statistical tests used, exact values of N, definitions of center, methods of multiple test correction, and dispersion and precision measures (e.g., mean, median, SD, SEM, confidence intervals; and, for the major substantive results, a measure of effect size (e.g., Pearson's r, Cohen's d)
- Report exact p-values wherever possible alongside the summary statistics and 95% confidence intervals. These should be reported for all key questions and not only when the p-value is less than 0.05.

**Group allocation**

- Indicate how samples were allocated into experimental groups (in the case of clinical studies, please specify allocation to treatment method); if randomization was used, please also state if restricted randomization was applied
- Indicate if masking was used during group allocation, data collection and/or data analysis
